# Mycotic Thoracic Aortic Aneurysm: Epidemiology, Pathophysiology, Diagnosis, and Management

**DOI:** 10.7759/cureus.31010

**Published:** 2022-11-02

**Authors:** Lekhya Raavi, Pankaj Garg, Md Walid Akram Hussain, Ishaq J Wadiwala, Nargis T Mateen, Mohamed S Elawady, Mohammad Alomari, Emad Alamouti-fard, Si M Pham, Samuel Jacob

**Affiliations:** 1 Cardiothoracic Surgery, Mayo Clinic, Jacksonville, USA

**Keywords:** mycotic thoracic aortic aneurysm, open repair, tevar, radiological findings, microbiology

## Abstract

Mycotic thoracic aortic aneurysm (MTAA) is an aneurysm of the aorta caused by infection of the vessel tissue through microbial inoculation of the diseased aortic endothelium. It is most commonly caused by bacteria. Rarely, it can be caused by fungi. However, viral aortic aneurysm has never been reported. Depending on the area and time period investigated, the infections organism discovered may vary significantly. Little is known about the natural history of MTAA due to its rarity. It is not known if they follow the same pattern as other TAAs. However, it is unclear whether MTAA follows a similar clinical course. The combination of clinical presentation, laboratory results, and radiographic results are used to make the diagnosis of MTAA. Treatment of MTAA is complex since patients frequently present at a late stage, frequently with fulminant sepsis, as well as concomitant complications such as aneurysm rupture. While medical treatment, including antibiotics, is recommended, surgery is still the mainstay of management. Surgery to treat MTAA is complicated and carries a high risk of morbidity and mortality and includes both open repairs and endovascular ones. In this review, we explore the etiology, pathogenesis, clinical presentations, diagnostic modalities as well as treatment management available for MTAA.

## Introduction and background

Mycotic aortic aneurysm (MAA) is a rare but deadly condition that results in aneurysmal dilatation of the aorta due to infection. It can be localized or involve an extensive area and can be slowly progressive or severe and rapidly growing. Pre-existing aneurysms can become secondarily infected, but aneurysmal arterial wall degeneration can also result from infection. The name mycotic aneurysm was coined by Osler to describe aneurysms associated with bacterial endocarditis as the aortic vegetations had the appearance of “fresh fungus vegetations”; however, the majority of MAAs are caused by bacteria. Different authors have used several different terminologies over time to address mycotic aneurysms that are misleading and without universal agreement. Recently, the term “mycotic aneurysm” has been replaced with “infective native aortic aneurysm” (INAA) to avoid confusion [1]. In western countries, MAA account for only 0.6% to 2.6% of cases of aortic aneurysms, but almost 13% of cases of aortic aneurysms in Asia, especially in East Asia [2-4]. MAA is not true but pseudoaneurysms as they lack all the layers of the aorta. Therefore, MAAs are significantly more prone to rupture than arteriosclerotic aneurysms and are associated with high mortality. It can involve any part of the aorta; however, the management becomes particularly challenging in the thoracic region. Early diagnosis and treatment with antibiotics and surgery are the keys to the successful management of MTAA [5,6]. Surgery remains the mainstay of management with open excision, debridement, and vascular reconstruction. Endovascular repair is also an acceptable alternative to open Surgery for MTAA repair, depending on the patient's medical condition, local practices, financial issues, and surgical and intervention team experience [7]. The endovascular repair for the aortic aneurysm was first reported in 1998, offering a less invasive treatment option for surgically high-risk patients [8]. Rarity and lack of data on MTAA led to their management, and outcomes were reported along with abdominal MAAs [9]. We designed a literature review with the aim of exploring the epidemiology, pathophysiology, diagnosis, and management of the mycotic aneurysm due to the significant diversity in the literature.

## Review

Epidemiology

In general, TMAA account for 30% of all mycotic aneurysms. There is a male predominance with a sex ratio of 3:1, and the average age of presentation is 65 years [10-12]. Endocarditis is the cause of the majority of arterial infections in the pre-antibiotic period [13].* *A higher prevalence of MTAA in males is likely due to a significantly higher prevalence of atherosclerotic aortic disease in males. Studies have reported that the atherosclerosis aortic wall is more vulnerable to microorganism colonization from concomitant bacteremia or adjacent infectious processes [14]. 

Earlier experience with MTAA, Parkhurst, and Decker [15] was reported in 1955 in an autopsy series of 22,792 cases performed in Boston between 1902 and 1951. In their series, MTAA was present in 12 cases (eight patients with descending thoracic aortic involvement and four with ascending aortic involvement), with a male-to-female ratio of 11:1. With the development of diagnostic tools and the evolution in the management, MTAA has been reported in patients. In one of the largest series on the MAA published by Weis-Müller et al. [16] in 2012 that included 36 cases of MAA (23 men and 13 women), with a mean age of 66.8 ± 8 years, MTAA was identified in only five patients (14%) and thoracoabdominal MAA an additional 13 patients (36%). In another case series by Hsu and Lin [17], the authors reported 32 patients with MTAA (24 men and eight women) over 12 years (from 1995 to 2007) with an age range of 50 to 88 years. In their series, aortic arch infection was present in 13 patients, proximal descending thoracic aorta infection in 10 patients, and distal descending thoracic aorta infection in nine patients. Another series by Müller et al. [18] included 33 patients (25 males and eight females) with MAA. The mean age of the patients was 64.3 years. In their series, descending thoracic aorta was involved in four patients, while thoracoabdominal MAA was present in two cases. In a retrospective review of patients with infectious aortic aneurysms by Miller et al. [19] from the Mayo Clinic, the authors reported 29 patients with MAA between 1976 and 1999. Nine patients in their series had MAA of the descending thoracic aorta. In another retrospective study by Cliff et al. [20], MTAA was found in three out of eight patients, all of whom were females.

Pathophysiology

The normal aorta in a healthy individual is very resistant to infection. However, patients who have pre-existing risk factors become predisposed to MTAA. 

Risk Factors

The thoracic aorta can get infected by organisms entering the aortic wall through the blood via intima or adventitia, lymphatics, vasa vasorum, or direct involvement from the adjacent structures. Risk factors that predispose to aortic infection include the following. 

Atherosclerosis and pre-existing aneurysm: Intact aortic intima is quite resistant to infection. However, atherosclerotic aortic disease denudes the intima and exposes the media to bacterial seeding. The diseased aorta is particularly vulnerable to typhoid and non-typhoid Salmonella species, and Salmonella is frequently isolated in MTAA due to atherosclerotic plaque [21]. Similarly, pre-existing aneurysms risk infection due to bacteremia or spread from a contiguous infection [22,23].

Antecedent infection: An antecedent infection in the vicinity of the thoracic aorta (e.g., pneumonia, endocarditis, peri-aortic lymphadenitis, purulent pericarditis, soft tissue infection, osteomyelitis, periodontal infection, and sepsis increase the risk of seeding of the thoracic aorta [24-27].

Impaired immunity: Healthy individuals are usually very resistant to aortic infection. However, immunosuppressive states, e.g., diabetes, alcoholism, chronic glucocorticoid therapy, chemotherapy, cirrhosis, chronic hemodialysis, posttransplant, human immunodeficiency virus (HIV) infection with acquired immunodeficiency syndrome (AIDS), and malignancy may predispose the patients to thoracic aortic infection [28-32].

Aortic injury: Arterial injury is an uncommon cause for MTAA as the thoracic aorta is usually well protected from injuries by the spine, sternum, and bony ribcage. However, iatrogenic injuries during cardiac surgery, aortic surgery, thoracic surgery, cardiac catheterization, or interventions, and blunt or penetrating traumatic injury to the thoracic aorta may predispose to infection [33,34].

Etiology

MTAA is most commonly caused by bacteria. However, rarely, it can be caused by fungi. However, a viral aortic aneurysm has never been reported. Organisms may seed the aortic wall by various mechanisms, including the contiguous spread of an infectious organism to the thoracic aorta, bacterial endocarditis emboli, systemic sepsis or cryptogenic/primary bacteremic, or bacteria directly introduced into the arterial wall due to trauma, which is primarily postprocedural [17,35-37]. Among them, bacteremic seeding of the aortic wall and contiguous spread are the most common mechanisms.

Bacteremic Seeding and Contiguous Spread

The vascular intima usually is highly resistant to infection. However, intimal integrity is jeopardized either due to iatrogenic or traumatic injury or congenital malformations (e.g., coarctation of the aorta), atherosclerotic plaque, or pre-existing aneurysm; bacteria may traverse into the deeper layers. Similarly, the contiguous spread can occur from pneumonia, periaortic lymphadenitis, suppurative pericarditis, and vertebral bacterial or tubercular osteomyelitis [38-43]. Once the thoracic aorta's local infection is established, it may result in suppuration, localized perforation, or pseudoaneurysm formation [44]. 

Septic Emboli

Septic emboli showering from the infective endocarditis can occlude the vasa vasorum of the thoracic aorta resulting in aortic wall infection and MTAA formation. Septic embolism is estimated to occur in 25%-50% of patients with infective endocarditis, but only about 1% to 5% develop MAA [45]. Due to its embolic nature, septic pseudoaneurysms are commonly multiple [46]. 

Direct Bacterial Inoculation

Although less common, direct inoculation of the bacteria in the thoracic aorta can occur at the time of vascular injury, e.g., iatrogenic, accidental, or assault (gunshot, stab). 

Microbiology

The pathogens detected may differ significantly depending on the region and period surveyed [28,21,47-50]. In the pre-antibiotic era, gram-positive cocci implicated in endocarditis (e.g., Staphylococcus, Streptococcus) were the most common organisms isolated from MTAA. However, in the post-antibiotic era, gram-negative organisms account for up to 40% of cases of MTAA, with Staphylococcus, Streptococcus, and non-typhoidal Salmonella species being the most common pathogens [17,51-63]. Furthermore, in western countries, Staphylococcus aureus (28%), Salmonella spp. (15%), and Pseudomonas aeruginosa (10%) are the most common causative organisms for MAA, while, in Asian countries, Salmonella has consistently been reported as the most common pathogen [64,65]. Salmonella, Typhimurium (serogroup B), Enteritidis (serogroup D), and Choleraesuis (serogroup C) are the most common species isolated [66,67].

Other gram-positive (e.g., Clostridium, Corynebacterium, and Enterococci), gram-negative (e.g., E. coli, Haemophilus influenzae, Proteus vulgaris, Yersinia enterocolica, Bacteroides fragilis, Burkholderia pseudomallei, Klebsiella pneumoniae, Coxiella burnetii, Campylobacter), Anaerobes (e.g., Bacteroides, Enterobacter, and Serratia, Bacillus Cereus), Syphilis, and Mycobacterium tuberculosis can also cause MTAA. True fungal MTAA is extremely uncommon and occurs due to contiguous spread from lung or disseminated systemic fungal infection in patients with immune suppression and diabetes mellitus. The most commonly isolated fungal species are Candida, Aspergillus, Scedosporium apiospermum, and Histoplasma. From 1966 to 1999, only seven cases of fungal causes of vascular aneurysms were reported (Table 1) [17,56,57,60,68-90].

**Table 1:** List of organisms responsible for mycotic thoracic aortic aneurysm.

Microbiology and different culture results				
Gram Positive	Gram Negative	Anaerobes	Fungal	Other
Staphylococcus	Escherichia coli	Bacteroides	Candida	Mycobacterium
Streptococcus spp	Haemophilus influenzae	Enterobacter	Aspergillus	Syphilis
Salmonella spp	Proteus vulgaris		Scedosporium apiospermum	
Clostridium	Yersinia enterocolica		Histoplasma	
Corynebacterium	Bacteroides fragilis			
Enterococci	Burkholderia pseudomallei [melioidosis]			
Bacillus cereus	Campylobacter spp			
	Klebsiella pneumoniae			
	Coxiella burnetii			
	Serratia			

Pathogenesis

An MAA occurs more frequently in an already-existing aortic aneurysm. Once the organism spreads to the aortic wall, it leads to further weakening and dilates the aorta. The pathogenesis of MTAA is similar to several other vascular diseases. Bacterial involvement of the aortic wall results in the generation of an inflammatory response with infiltration of neutrophils and lymphocytes in the aortic wall. These blood cells secrete various inflammatory cytokines, including Metalloproteinases (MMPs) and neutrophil gelatinase-associated lipocalin (NGAL), to kill the bacteria and contain the infection. MMPs break down the extracellular structural proteins and play a key role in tissue remodeling and the pathogenesis of MTAA. NGAL prevents the breakdown of MMPs and perpetuates the destruction of the aortic walls by MMPs. Studies have shown that increased MMP activity is associated with a higher risk of aortic rupture [91].

**Figure 1:**
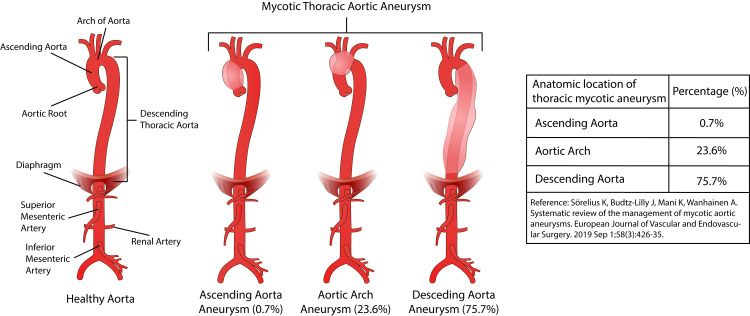
Anatomical location of thoracic mycotic aneurysm. Most of the MTAA occur in the descending thoracic aorta (75.7%) while the least occur in the ascending aorta (0.7%). The table on the right describes the proportion of MTAA occurring in different anatomic locations of the thoracic aorta. (Table data from a study published by Sorelius et al. [92]; diagram designed by Md Walid Akram Hussain.)

In contrast to true aneurysms, most MTAAs are pseudoaneurysms. The bacterial infection leads to the partial or complete destruction of the aortic wall, and the aneurysm is mainly covered by fibrous tissue [19]. As per Laplace law, arterial pulse pressure against a compromised aortic wall results in increased wall tension and aneurysmal enlargement of the aortic wall. Animal studies on MAA revealed that bacterial infection initially weakens the adventitial layer before spreading to the media [35]. The aneurysm's shape depends upon the site and type of aortic wall involvement. In the case of circumferential involvement or MTAA in previous aortic aneurysms, it is fusiform, while in cases of contiguous spread and septic embolization, the aneurysm is usually saccular. MTAA are at significant risk of rupture due to increased wall stress; aortic rupture can occur without aneurysm formation, termed infective aortitis. The prevalence of thoracic mycotic aneurysm varies depending on the location, with descending aorta having the highest prevalence of 75.7% and ascending aorta with the lowest of 0.7% (Figure 1) [92].

Histopathologically, MTAA is characterized by infection and signs of arterial wall damage, including inflammation, necrosis, abscesses, thrombosis, and the usual presence of bacteria [19,93]. Syphilitic aortitis is an exception that develops due to Treponema pallidum invasion of the aortic wall and lacks the conventional signs of bacterial infection [94-96]. Syphilitic aortitis develops due to obliterative endarteritis of the vasa vasorum and necrosis of the elastic fibers and connective tissue in the aortic media; therefore, it is not considered a part of mycotic aneurysms [97].

Natural history, clinical features, and screening

Due to rarity, little is known about the natural history of MTAA. It is uncertain whether they follow the same pattern as other thoracic aortic aneurysms (TAA). For TAA, the risk of rupture increases with increasing size, reaching almost 7% annually once the aneurysm is TAA >6.0 cm in size [98]. Clinical presentation of MTAA is frequently nonspecific and depends upon the site and severity of infection, comorbidities, and size of the aneurysm [99]. The most common presenting symptoms are fever (75%), chest and back pain (60%), abdominal pain (20%), and chills (16%). Some patients may even be completely asymptomatic [100-103]. In the study of seven patients with MTAA, Johnstone et al. [104] demonstrated that all the patients were symptomatic at presentation, with pain being the most common symptom. In addition, three patients presented with fever (>101°F), two presented with hemoptysis from an aortobronchial fistula, and two complained of fatigue. In a retrospective study of 33 patients with MTAA by Muller et al. [18], authors reported that 79% of patients had signs of infections (raised C-Reactive protein [CRP] and lymphocyte count), 48% of patients had a fever, and 24% of patients had positive blood cultures. Further, 76% of patients complained of regional pain, such as severe back or chest pain, four patients with thoracoabdominal MAA had respiratory failure, and two were intubated and mechanically ventilated preoperatively.

Local expansion of an MTAA may result in compressive symptoms such as dysphagia, dyspnea, hoarseness, cough, and superior vena cava syndrome [105,106]. Patients with an arch or upper descending MTAA may develop Ortner's syndrome (Cardio-vocal Syndrome) due to the compression of the recurrent laryngeal nerve between the aortic arch and the left pulmonary artery. Rarely, MTAA may develop an aortoesophageal fistula with midthoracic pain, fever, and hoarseness of voice [101].

If the diagnosis is delayed, MTAA may also present devastating complications such as rupture and bleeding. In a study by Steverlynck et al. [106], ~60% of patients with MTAA presented with rupture, and surgical survival in these patients was as low as 35%. MTAA may rupture and bleed into the adjacent structure, e.g., ascending aortic MTAA in the pericardial cavity, descending thoracic MTAA in the pleural cavity, or rarely, into the trachea, bronchi, or esophagus [101]. As the symptoms are primarily nonspecific, especially in patients who are IV drug users, immunocompromised, have undergone invasive procedures, and those with a history of endocarditis, patients may just present with a fever of unknown origin. Therefore, in high-risk patients, there should be a high index of suspicion, and the patient should be evaluated early for MTAA before developing complications like sepsis, thrombus formation, bleeding, or rupture [50].

Due to the presentation of nonspecific symptoms and the unavailability of screening tests, it becomes challenging to suspect MTAA. Therefore, in symptomatic patients with predisposing factors, e.g., atherosclerotic risk factors, immunosuppression, and evidence of concurrent infection (infective endocarditis, pneumonia, endocarditis, purulent pericarditis, soft tissue infection, osteomyelitis); MTAA should be included as a differentials diagnosis and patient should be evaluated early to prevent devastating complications and reduce morbidity and mortality.

Diagnosis

The lack of specific presenting features, inability to palpate the aneurysm, and insufficiency of sensitivity of chest x-ray, diagnosis of MTAA is frequently missed. Presently, no single investigation is sufficient to diagnose MTAA, and there is no available algorithm for diagnosing MTAA [107]. Therefore, the diagnosis of MTAA is based on a combination of the following criteria: (a) clinical presentation (pain, fever, evidence of concomitant infection, an elderly patient with cardiovascular disease, and/or immunosuppressive state), (b) laboratory findings (raised inflammatory parameters including CRP, leukocytosis, and positive blood culture); and (c) radiological findings on contrast-enhanced computed tomography (CECT) or magnetic resonance imaging (MRI) of the chest.

Laboratory Findings

In patients with MTAA, inflammatory markers, such as erythrocyte sedimentation rate (ESR), CRP, and leukocytosis, are frequently elevated [108]. The diagnosis is further supplemented by positive blood culture. Blood cultures are positive in 50% to 85% of patients with a mycotic aneurysm. A commonly single organism is isolated on blood culture; however, multiple organisms can be isolated in 8% of patients, and no pathogen may be isolated in 25% of patients [24]. Non-typhoid Salmonella, Staphylococcus, Campylobacter, and Streptococcus are the most commonly identified pathogens [109], but E. coli, Mycobacteria, and Bacteroides species have also been reported. Isolation of infective microorganisms and confirmation of their antibiotic sensitivity helps in antimicrobial selection. Bacterial isolation, however, may not always be possible due to difficulties in culture or prior antibiotic treatment [48]. Organism identification can be improved by polymerase chain reaction. *Imaging*

Precision diagnostic data from a combination of contrast-enhanced computed tomography (CECT) chest and angiographic imaging is required to diagnose and plan appropriate and timely surgical therapy [50]. The electrocardiographic gating technique can further improve the accuracy of CECT imaging by detecting the more subtle aortic lesions by eliminating the pulsation motion artifacts in the descending thoracic aorta [106,110]. Multidetector CECT coronary angiography is currently the imaging modality of choice for evaluating suspected infected MTAA [3]. CECT chest imaging features of an infected TAA are contrast-enhancing saccular-shaped aneurysms associated with periaortic soft tissue mass, edema, or abscess. The rapid change in size or shape of the aneurysm in follow-up CT studies is a crucial CT feature of MTAA. However, peri-aortic gas is a rare but helpful clue for the possible diagnosis of MTAA with aorto-enteric fistula. Calcification of MTAAs is uncommon compared to uninfected aneurysms. While a CECT scan of the chest is the preferred initial imaging modality for aortic aneurysms, contrast-enhanced MRI with angiography of the chest is an acceptable alternative in patients with contraindications to CECT [111]. Although more invasive, digital subtraction angiography can provide the same information [99]. Nuclear scans, including positron emission tomography (PET), have primarily been used to detect aortic graft infection [112]. Infectious endocarditis patients may have multiple aneurysms, necessitating more extensive angiography and imaging studies [50]. To assess disease activity, gallium scanning and 18F-fluorodeoxyglucose positron emission tomography (FDG-PET) may be used [113]. The value of PET and granulocyte scintigraphy has not been appropriately evaluated but could probably aid in diagnostics in uncertain cases [107].

Management

**Figure 2:**
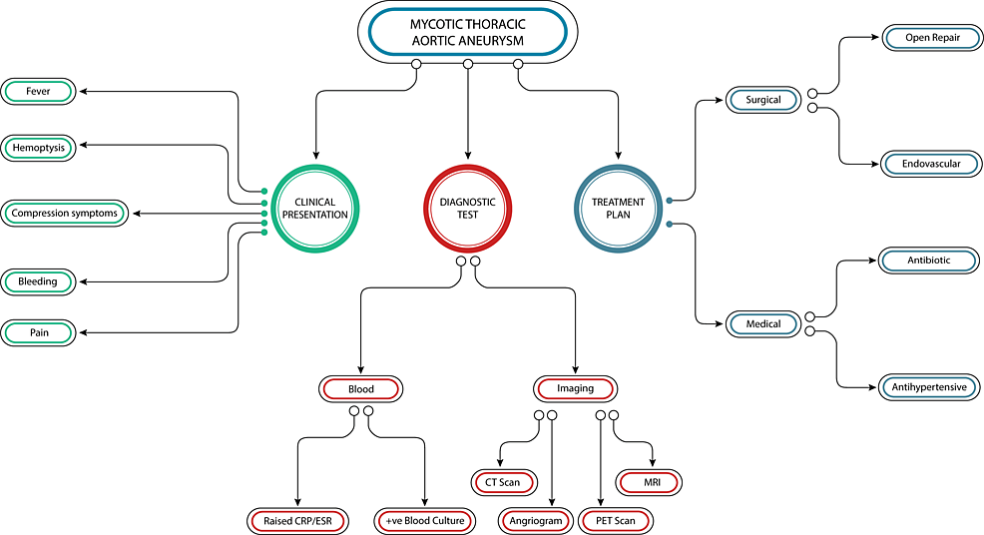
Diagnostic evaluation of mycotic thoracic aortic aneurysm. The figure above outlines the management plan for diagnosing and treating a mycotic thoracic aortic aneurysm. The clinical presentation can vary from fever, and pain, to hemoptysis. For diagnosis, both blood work and imaging modalities are used. Treatment options include medical and surgical interventions. Medical treatment mainly involves antibiotics and antihypertensive medications, while surgical options include open surgery and minimally invasive options (endovascular options). (Figure designed by Md Walid Akram Hussain.)

There are many challenges associated with treating (MTAA) since the patient often presents at a late stage and usually with fulminant sepsis and other complications, including rupture of the aneurysm. Surgery is the mainstay of management, while medical management using antibiotics is supplementary and not a replacement for surgery. MTAA is a surgical urgency and should be addressed as soon as the diagnosis is made while the appropriate antibiotic therapy is administered [1]. Complete excision of the infected aorta is crucial to the curative treatment. Surgical repair options include both open repair as well as endovascular therapy (Figure 2). 

Medical Management

There is limited data on the role of exclusive treatment with antibiotic therapy in patients with MTAA. In a few studies where patients were exclusively treated with antibiotics, in-hospital mortality was very high, ranging from 75% to 100%, with all deaths attributed to the rupture of the aneurysm [103,114]. Perioperative and postoperative use of antibiotics for MTAA is a part of the standardized protocol; still, the consensus is lacking on the optimal duration of antibiotics [1,115]. A two- to six-week of preoperative antibiotic course has been advocated as a standard practice unless the patient's condition mandates emergency surgery [17,48,116,117]. Kan et al. [116] in their study reported that preoperative antibiotic treatment for more than three days reduced the odds ratio of aneurysm-related mortality to 0.2 (95% CI] 0.04-0.96; p = 0.053).

Similarly, Sörelius et al. [118] reported that postoperative antibiotic for more than six months was associated with a hazard ratio of 0.36 (95% CI 0.18-0.74, p = 0.005) for late mortality. However, the duration of postoperative antibiotics is highly variable depending upon the severity of the disease, associated organism, and immune status of the patient and may vary from complete absence to four to six weeks, to three to six months, to six to 12 months, to lifelong and should be individualized [119-123] immunosuppressed patients and patients in whom biochemical parameters of inflammation fail to normalize may require a longer antimicrobial course [100,102]. Rarely, however, have patients survived antibiotic treatment alone without surgery. For example, Yano et al. [75] reported a case of an 81-year-old patient suffering from MTAA managed with antibiotics alone for three months without surgical intervention with complete recovery. Still, medical management should be reserved for patients who are old, moribund, or have multiple comorbidities that increase the risk of mortality.

Apart from antibiotics, other supportive measures for MTAA are similar to those used for aortic dissection. The main goal for both symptomatic and asymptomatic patients is to normalize blood pressure as blood pressure control reduces the wall stress, stabilizes the extracellular matrix of the aorta, and reduces the possibility of aneurysm expansion and rupture. Beta-blockers are the first-line agent for controlling blood pressure management in MTAA [106].

Surgical Management

The surgical treatment of MTAA is challenging and entails a substantial risk of morbidity and mortality. This comprises both endovascular and open repairs. Traditionally, most centers preferred open repair, but after 2007, the use of thoracic endovascular aortic repair (TEVAR) has steadily increased. The mortality rate following MTAA repair was dependent on the segment of the aorta which was involved as well as the approach of surgical repair. The 30-90-day mortality rate for the repair of MTAA involving descending thoracic aorta was estimated at 15% for TEVAR and 7%-20% for open surgical repair (OSR) [5,17,123]. Similarly, mortality for the repair of MTAA involving the arch of the aorta at 30-90 days ranged between 25% for TEVAR and 10%-60% OSR [17,123,124].

Unless contraindicated, all patients with MTAA should undergo surgery as soon as possible, regardless of the size and site of the aneurysm. However, some surgeons advocate algorithm-based management based on the hemodynamic stability of the patient and the progression or remission of symptoms. For hemodynamically stable patients who respond well to immediate medical treatment, delayed surgical repair should be offered to maximize antibiotic benefit. However, hemodynamically unstable patients, in whom the pain does not remit or has a progression of the aneurysm, should undergo prompt surgery [17,103,114,116,117]. 

Kan et al. [125] reported that patient-related factors like advanced age, non-Salmonella infection, leukocytosis, aortoenteric fistula, and shock are significant predictors of aneurysm-related morbidity and death, and it is not related to the technique of repair, e.g., TEVAR or open surgery procedures. Stevelynck et al. [106], in their study, outlined several ways in which the TEVAR procedure in mycotic aneurysms can be improved. This included: 1. Use broad-spectrum antibiotics as soon as the patient is suspected of having MTAA. 2. Use of antibiotic-coated endoprosthesis. 3. Surgical debridement or percutaneous drainage may also help eliminate the source of infection. 4. Extended use of antibiotic therapy in the postoperative period (D) [106].

Open Surgical Resection (OSR)

Open surgical resection consists of complete excision of the aneurysm and extensive debridement of the infected tissue, followed by establishing the continuity with complete revascularization. This can be performed using various techniques (in-situ reconstruction or extra-anatomic bypass). These can be divided into (a) Excision of aneurysm followed by ligation with no arterial reconstruction: This technique is suitable for a peripheral vascular mycotic aneurysm. However, it is rarely used for the management of MTAA. (b) Excision and patch repair of the aneurysm: this technique is suitable for saccular MTAA. In this technique, aneurysmal tissue of excised and debrided to normal aortic tissue, and the defect is repaired with a homograft aortic patch or Dacron patch repair with or without reinforcement with omentopexy [84,126]. (c) Excision followed by interposition graft insertion: This technique is the mainstay of managing fusiform aneurysms involving any part of the thoracic aorta. In this technique, an aneurysm is excised completely with debridement of infected and necrotic tissue, and continuity is established with a Dacron graft. Additional procedures that be required depending upon the site are aortic root replacement with Bentall, coronary artery bypass grafting, valve-sparing aortic root replacement, ascending aortic replacement, re-implantation of Arch vessels, and re-implantation of intercostal vessels [84]. (d) Extra-anatomic Bypass: In this technique, continuity is established between the aortic segment proximal and distal to the MTAA by using a Dacron graft passed in an extra-anatomical position. This surgical technique is suboptimal as the infected tissue remains in situ and is at risk of progression and rupture. This technique is more commonly used for a peripheral vascular mycotic aneurysm. However, in MTAA, this technique can be used for patients with descending TAA where aneurysmal tissue or left pleural space is frozen due to previous surgery. It is impossible to excise the infected tissue safely. Further, it can be a technique of choice in patients unsuitable for extensive surgery due to their physical condition or co-morbidities. 

The above techniques come with serious complications sometimes. The most commonly reported complications after extra-anatomic bypass were blowouts of the aortic stump in 2% [122,123], graft occlusions in 31% [114], and claudication after graft stenosis in 36% [114,122]. Complications following in-situ reconstruction include dehiscence of anastomosis and bleeding in 1% of patients and no reported data on graft occlusion or claudication [118,120,127].

Thoracic Endovascular Aortic Repair (TEVAR)

The advent of endovascular therapy has gradually replaced the open surgical repair of thoracic and abdominal aortic aneurysms. Therefore, it is not surprising that endovascular techniques are being used more frequently as an initial temporizing measure and definitive restorations of MTAA. The first report on TEVAR for MTAA was published by Semba et al. [8] in 1998. He operated on three patients with MTAA with TEVAR. Since then, TEVAR has been used more frequently to manage MTAA. It may seem contradictory to fundamental surgical dogma to use an endograft in an infected area. However, the results of numerous studies have shown comparable outcomes with the endovascular approach. In a recent survey by Sörelius et al. [128], authors reported that a large proportion of MAAs in Sweden are being treated with endovascular repair with short- and long-term survival rates similar to open surgery.

Further, the endovascular approach is more attractive as it offers several advantages over open surgical repair, including less physiologic stress, reduced blood loss, obviating the need for large thoracotomy or sternotomy incision, cardiopulmonary bypass, aortic cross-clamping, full anticoagulation, intubation, and single lung ventilation. Therefore, it reduces the risk of surgery-related morbidity and mortality, respiratory and renal failure, extended extremity, or organ ischemia [129].

The main disadvantage of TEVAR is placing a foreign body in an infected field, which contradicts general surgical principles as it may serve as a reinfection nidus [125]. Furthermore, TEVAR treatment does not include local debridement of the infected field. Still, the reported rate of reinfection is relatively low. Indeed, a study reported that 81.2% of patients did not suffer from reinfection even after two years [130]. One of the main complications of TEVAR is endoleak, and one study demonstrated a high incidence of endoleak, with around 18.5% suffering from it [130]. In the case of infection after TEVAR placement, owing to the lack of direct tissue culture in TEVAR, no organism may be specifically identified, and broad-spectrum antibiotic treatment should be initiated. While TEVAR is safe, one study has suggested that in the hands of an expert surgeon, open surgical repair is still the best option, with a mortality of less than 10% achievable [124].

Hybrid Repair

Hybrid repair combines open and endovascular repair, either a staged approach or combined as a single-stage procedure. Arch MTAA aneurysm can be exteriorized completely with a covered stent graft after surgically bypassing all the arch vessels to the ascending aorta (debranching). This technique avoids circulatory arrest and extensive arch reconstruction. Similarly, MTAA involving the distal arch or proximal descending thoracic aorta can be operated by a hybrid technique by first bypassing the left subclavian artery with a carotid artery to subclavian artery bypass and later exteriorizing the mycotic aneurysm with a covered stent. This technique completely avoids sternotomy and cardiopulmonary bypass. For thoracoabdominal MAAs, mycotic aneurysms can be eliminated with a hybrid technique. Usually, the first part of the repair involves an open surgical procedure in which the visceral vessels are bypassed, and an endovascular stent graft is used to exclude the aneurysm completely. As these repairs avoid thoracotomy, supra-coeliac aortic cross-clamping, and left or full heart bypass, the chance of the patient suffering from ischemia-reperfusion injury is reduced [131].

## Conclusions

MTAA is rare and presents with vague symptoms. It often results in devastating consequences with treatment options that have high mortality. As a result of its rarity, most studies are small retrospective single-center studies. The TEVAR appears to be associated with improved short-term survival, without late disadvantages, compared with the OSR. This suggests that endovascular repair may be a suitable alternative to Open surgical repair. To get the best results, MAA treatment must always be planned and tailored to the individual patient's needs, which makes general treatment recommendations useless for each individual. This is dependent on surgeon skills as well as the availability of facilities. Collaboration among multiple institutions is also crucial to improving surgical care.
